# The epistemic harms of empathy in phenomenological psychopathology

**DOI:** 10.1007/s11097-023-09930-1

**Published:** 2023-08-12

**Authors:** Lucienne Spencer, Matthew Broome

**Affiliations:** 1 Institute of Mental Health, University of Birmingham, Birmingham, UK; 2 Birmingham Women’s and Children’s NHS Foundation Trust, Birmingham, UK

**Keywords:** Empathy, Jaspers, Epistemic injustice, Epistemic objectification, Transformative experience, Phenomenological, Psychopathology

## Abstract

Jaspers identifies empathic understanding as an essential tool for grasping not the mere psychic content of the condition at hand, but the lived experience of the patient. This method then serves as the basis for the phenomenological investigation into the psychiatric condition known as ‘Phenomenological Psychopathology’. In recent years, scholars in the field of phenomenological psychopathology have attempted to refine the concept of empathic understanding for its use in contemporary clinical encounters. Most notably, we have Stanghellini’s contribution of ‘second-order’ empathy and Ratcliffe’s ‘radical empathy’. Through this paper, we reject the pursuit of a renewed version of ‘empathic understanding’, on the grounds that the concept is fundamentally epistemically flawed. We argue that ‘empathic understanding’ risks (1) error, leading to misdiagnosis, mistreatment and an overall misunderstanding of the experience at hand, (2) a unique form of epistemic harm that we call ‘epistemic co-opting’ and (3) epistemic objectification. To conclude, we propose that empathic understanding ought to be replaced with a phenomenological account of Fricker’s virtuous listening.

## Introduction

In his magnum opus ‘General Psychopathology’, published in 1913, the renowned philosopher and psychiatrist Karl Jaspers proclaimed: ‘Rational understanding is merely an aid to psychology, empathic understanding brings us to psychology itself’ (Jaspers, 1997: 304). Jaspers identifies empathic understanding as an essential tool for grasping not the mere psychic content of the condition at hand, but the lived experience of the patient. This method then serves as the basis for the phenomenological investigation into the psychiatric condition known as ‘Phenomenological Psychopathology’.

In recent years, scholars in the field of phenomenological psychopathology have attempted to refine the concept of empathic understanding for its use in contemporary clinical encounters. Most notably, we have Stanghellini’s contribution of ‘second-order’ empathy (Stanghellini, 2013) and Ratcliffe’s ‘radical empathy’ (Ratcliffe, 2012). Through this paper, we reject the pursuit of a renewed version of ‘empathic understanding’, on the grounds that this aspect of the methodology is prone to error and fundamentally ethically flawed.

We begin by examining Jaspers’ account of ‘empathic understanding’ and its significance within the wider contexts of phenomenological psychopathology. We then highlight common criticisms launched at ‘empathic understanding’, before highlighting attempts to develop the concept. Nevertheless, we conclude that it is impossible to adopt Jaspers’ concept of empathic understanding without inheriting the epistemically unjust attitudes that accompany it. We argue that ‘empathic understanding’ risks (1) error, leading to misdiagnosis, mistreatment and an overall misunderstanding of the experience at hand, (2) a unique form of epistemic harm that we call ‘epistemic co-opting’ and (3) epistemic objectification. Finally, we propose that we ought to do away with the concept of empathic understanding altogether and replace it with a phenomenologically infused kind of ‘virtuous listening’ that reinstates the epistemic agency of the psychiatric patient.

## Empathic understanding

### Empathy

Philosophers have been captivated by the concept of empathy since the term’s relatively recent introduction into our vocabulary via the German ‘*Einfühlung’* in 1873. It is a term that continues to defy easy definition, and its characterisation seems to depend on the intention of the user. Driven by the ‘theory of mind’ debate, scholars attempted to theorise empathy in order to address the question ‘how, and to what extent, can we come to understand others?’. Contenders for theories of empathy include simulation theory, theory theory, and the argument from analogy (Zahavi, 2010). Many of the significant contributors to the richer concepts of empathy belong to the field of phenomenology, including Scheler (1923), Stein (1917), and Husserl (1962). These phenomenological accounts grapple with the interpersonal dimension of empathy, empathy as an intentional experience and empathy as a means of experiencing the other (Zahavi, 2014).

Since its introduction to psychiatry by Theodore Lipps, empathy has played a significant role in mental health discourse (Lipps, 1907). Although Lipps evoked the term ‘empathy’ in order to identify our capacity for understanding others on a primordial level, in public mental health literature, the term has been appropriated as a corrective tool for bad ethical practices in psychiatric care (Kitwood, 1997; Mercer et al., 2004; Spiro, 2009; Gelhaus, 2012; Jütten et al., 2019) (Barrera et al., 2023). In these contexts, empathy takes the form of a compassionate attempt to relate to the experience of the person with a mental health condition. Through such empathy, the healthcare professional is perceived to cultivate virtuous interpersonal practices of fostering trust, demonstrating respect and adopting a sympathetic attitude. Although some proponents of these arguably shallow accounts of empathy recognise that practising empathy improves our understanding of a given condition, its primary function is to ‘enhance the doctor–patient relationship and to improve patient enablement, and patient and doctor satisfaction in clinical encounters’ (Mercer et al., 2004: 700). While some scholars challenge this account of empathy as a virtue in the medical encounter (Gardner, 2015; Stefanello, 2022), the dominant view that empathy is necessary for good clinical practice persists.

Our goal in this paper is not to weigh in on the competing accounts of empathy in philosophy, or to challenge the ‘care-based’ accounts of empathy popular in public health care. For the purpose of this paper, we specifically target the use of Jaspers’ ‘empathic understanding’ in contemporary approaches in phenomenological psychopathology. We hope to show a serious need to re-evaluate this concept, on both epistemic and ethical grounds.

### Jaspers’ empathic understanding

Karl Jaspers infuses the phenomenological tradition of early Husserl with the psychology of his contemporaries, such as Wilhelm Dilthey, Max Weber, and Georg Simmel, to form a revolutionary approach to psychiatric practice known as phenomenological psychopathology. In 1912 Jaspers writes “The Phenomenological Approach in Psychopathology” (1968), which acts as a blueprint for Jaspers’ ‘General Psychopathology’. In this paper, Jaspers begins by distinguishing between the objective and the subjective symptoms one can examine in a psychiatric patient. Objective symptoms can be observed on the surface and deduced through sense perception and ‘rational thought’ (Jaspers, 1968: 1314). Objective symptoms include (1) ‘concrete events that can be perceived by the senses’ (e.g. physical gestures and speech expression), (2) ‘all measurable performances’ (e.g. whether the patient has the capacity to work, or learn, or retain memory) (3) ‘the rational content of what the patient tells us’ (e.g. reports of delusion) (ibid.). These objective symptoms were the main focus of the psychotherapist in Jaspers’ day (and arguably continue to dominate modern psychiatry).

In contrast, Jaspers identifies that the psychiatric patient also has subjective symptoms that are not so easily assessed. Inspired by early Husserl, Jaspers understands subjective symptoms to be the elusive inner life of the psychiatric patient. For Husserl, the object of phenomenology is the pure description of how phenomena appear to consciousness. In the words of Laverty, ‘Phenomenology… was seen as a movement away from the Cartesian dualism of reality being something ‘out there’ or completely separate from the individual’ (Laverty, 2003: 23). Instead, Husserl posits that the subject and the world are irrevocably intertwined. While the Cartesian view, according to Husserl, presented consciousness as ‘empty of content’, a phenomenological approach reveals the rich content of consciousness (Husserl, 1970: 155). This can be understood as the emotional, temporal, spatial, and intentional style of one’s embodied experience in the world.

Through Husserl, Jaspers claims ‘I found confirmed what was already at work in me: the urge to the things themselves. In a world full of prejudices, schematisms, conventions, this was at the time like a liberation’ (Jaspers, 1958: 386)^1^. In finding Husserl, Jaspers enthusiastically put aside the traditional methods and narrow frameworks of psychopathology to reconstruct it anew. Rather than ‘objective phenomena’, Jaspers sought to excavate the subjective phenomena of the patient’s psychiatric condition- their lived world experience. How does one access subjective phenomena? Unlike objective symptoms ‘subjective symptoms cannot be perceived by the sense-organs, but have to be grasped by transferring oneself, so to say, into the other individual’s psyche’ (Jaspers, 1968: 1314). Jaspers calls this ‘empathic understanding’^2^.

Before we consider the role of ‘empathy’ let us briefly reflect on ‘understanding’ in psychiatric healthcare. The medical encounter begins with an important question: in what way are you feeling unwell? In somatic medicine, this question triggers the start of a diagnostic process that involves the combination of the patient’s testimony and a direct physical examination of the physical malady. However, in psychiatric healthcare, this question has a unique quality. This question is the start of an excavation into the internal and ineffable mental life of the psychiatric patient: ‘Psychopathology functions as a bridge between the human and clinical sciences, providing the basic tools to make sense of mental suffering’ (Stanghellini & Broome, 2014: 170). While Jaspers suggests that aspects of the psychic life are readily available through objective symptoms, the life-world of the patient is not so easily accessible. For this reason, ‘understanding’, ‘perception of meaning’ or ‘*Verstehen*’ plays a central role in Jaspers work (Jaspers, 1997:27). He recognises that psychiatric healthcare is essentially a hermeneutical endeavour, an exercise in understanding and meaning-making.

For those conditions that result in profoundly unusual experiences, there is no straightforward, universal translation for any psychiatric experience. Rather, the healthcare professional must consider the vast landscape of interpretations the patient may call upon and act as ‘the investigator of meaning’ (Jaspers, 1997: 314). For instance, an experience of hallucination may present itself as terrifying and uncanny for one person. It might elicit a bodily reaction (such as cowering), stimulate particular beliefs (‘I am in danger’) and will induce particular feelings connected with this meaning (such as despair). Alternatively, a hallucination may be experienced as a positive, spiritual event, eliciting entirely different bodily responses, beliefs and feelings. It may not be interpreted as an illness at all. As such, phenomenological psychopathology is not just about identifying and supporting the treatment of a psychiatric ‘illness^3^’; it is about exposing the unique meaning structures of that psychiatric condition for the patient.^4^ Moreover, understanding for Jaspers goes beyond merely grasping a patient’s experience. He identifies understanding as a form of ‘knowledge that can be communicated, investigated and argued about’ and equal, necessary, and parallel to causal understanding (Jaspers, 1968: 1315).

Jaspers observes that one manner in which the ‘psychic life’ could be interpreted is through a ‘genetic understanding’: ‘The understanding of the meaningful connections between one psychic experience and another, the “emergence of the psychic from the psychic”’ (Jaspers, 1968: 1322). ‘Genetic understanding’ could be construed as a Diltheyan hermeneutic technique, whereby ‘the examiner acquires an understanding of how a mental phenomenon arises from another mental phenomenon in a meaningful way’ (Häfner, 2015: 20)^5^. For example, ‘attacked people become angry and spring to defence, cheated persons grow suspicious’ (Jaspers, 1997: 302). It also has links to modern-day neuroscience, as, drawing on Dilthey, Jaspers also recognises genetic understanding as ‘the ascertainment of the physical basis of psychic processes’ (Jaspers, 1968: 1323). For Jaspers, this form of understanding is distinct from the phenomenological approach to understanding; while phenomenology is a ‘cross-sectional mode of inquiry’ genetic understanding is a ‘longitudinal approach’ (Häfner, 2015: 20). That is not to say he thought we ought to discard ‘genetic understanding’. Jaspers acknowledged that it held a valuable place in ‘penetrating the extra conscious depths’(Jaspers, 1968: 1323). We require both forms of understanding in order to properly access the psychiatric illness of the patient, as Jaspers combines the early, static phenomenology of Husserl^6^ with Diltheyan hermeneutics^7^.

Genetic understanding is explicitly linked with empathic understanding for Jaspers. Through empathic understanding, the psychiatrist aims to place themselves in the position (the lived situation) of the patient, to the extent that they can recreate their lived experience: ‘we sink ourselves into the psychic situation and *understand genetically by empathy how one psychic event emerges from another*’ (Jaspers, 1997: 301). It is important to note that the psychiatrist may in fact have experienced, or may be experiencing, the same ‘psychic situation’ as the patient. There is a danger of perpetuating an ‘us and them’ dynamic in mental health literature between the clinician and the patient, as though the clinician were an abstract being, devoid of illness experiences. Indeed, such lived experiences may be the cause for the clinician to pursue a career in psychiatry. In cases where the clinician has had a lived experience of the condition in question, this step of empathic understanding would not be necessary, as they would already have the experiential knowledge required. Clinicians who need to exercise empathic understanding are those without the requisite lived experience.

Jaspers is careful to draw a clear distinction between empathy and sympathy here. He recognises that sympathy is valuable as a virtue in clinical practice because ‘completely dispassionate observation misses the essence of things’ (Jaspers, 1997: 22). However, while it is a catalyst for understanding, sympathy is not a means of understanding in itself. Its predominant role in psychiatry is to gratify, not to understand. The goal of empathy in psychiatry, on the other hand, is to ‘gain an essentially personal, indefinable and direct understanding, which, however, remains for him a matter of pure experience, not of explicit knowledge’ (Jaspers, 1968: 1315).

This ‘empathetic understanding’ is not easily accomplished. Jaspers acknowledges that it requires both practice and the ability to perform an act that very closely resembles the Husserlian phenomenological reduction or ‘bracketing’^8^. Before empathic understanding can occur, the clinician must pursue an ‘isolation of phenomena’, a suspension of ‘all outmoded theories, psychological constructs or materialist mythologies of cerebral processes’ and ‘basic constructs or frames of reference’(Jaspers, 1968: 1315–1316). This bracketing would evidently include the taxonomy and classification pre-established in psychiatry, as well as all inherited, obsolete psychological theories that may influence the psychiatrist. More generally, Jaspers calls for a suspension of how we assume ‘psychic events’ to be through an ‘unprejudiced direct grasp’ of the subjective phenomena (ibid.). Once this ‘freedom from preconception’ has been achieved, the clinician can access the lived experience of the patient through empathic understanding (Jaspers, 1968: 1316).

Jaspers oscillates between empathic understanding being a direct form of access to an indirect form of access of the patient’s experience. On the one hand, he describes empathic understanding as transferring oneself into the Other so the psychiatrist can directly access the patient’s life world. As such, through a unique form of perception, empathy ‘always leads directly into the psychic connection itself’ (Jaspers, 1997: 304). On the other hand, Jaspers also claims ‘those psychic experiences and phenomena which patients describe to us…only become accessible to us at second-hand’ (Jaspers, 1968: 1313). He states: ‘Direct, accessible psychic experiences are like the foam on the sea’s surface. The depths are inaccessible and can only be explored in an indirect and theoretical way’ (Jaspers, 1997: 10). To grasp the role of empathic understanding within the methodology of phenomenological psychopathology, we require a clear understanding of the level of access the clinician can assume through empathy.

On phenomenological grounds, Jaspers’ account of direct empathic understanding as ‘transferring oneself…into the Other individual’s psyche’ is initially difficult to contend with (Jaspers, 1968: 1313). Setting aside the practicality of such a task, inhabiting the consciousness of the Other would entail no clear distinction between an existence as myself from an existence of all other entities in the world; from an ‘I’ to the existence of an ‘Other’. Such a view is impossible to imagine as the concept of ‘Other’, pertaining either to objects or other people, is a meaningful one. We have confidence in our existence as an ‘I’ and can clearly distinguish ourselves from other objects and subjects in the world because our experience of ‘I’ is essentially limited to our own embodied experience:

when I experience myself, and when I experience others, there is in fact a common denominator. In both cases I am dealing with *embodiment* and one of the features of my embodied subjectivity is that it, per definition, entails acting and living in the world. (Gallagher & Zahavi, 2012: 206).

On the surface, it would seem that ‘empathic understanding’ misses the embodied, situated nature of consciousness, not just as another being-in-the-world but as a distinct lived body.

To address this problem, it is worth fleshing out Jaspers’ concept of direct empathy with Husserl’s own account. Husserl^9^ advocated for empathy as a quasi-direct experience of the Other: ‘the intentionality in one’s own ego that leads into the foreign ego’ (Husserl, 1962: 321). To be clear, Husserl does not suggest that it is possible to genuinely constitute Others in the manner that they constitute themselves. Rather, we embrace the Other into our subjectivity in the same way we embrace other aspects of the world- through intentionality. The pen is part of my bodily possibility to write; the tea is part of my bodily possibility to drink, and so on. Similarly, we embrace the Other as a possibility for interaction. Much like Jaspers, Husserl compares empathy to a form of perception. When I see the other smile, I directly perceive their expression of joy. No inference is necessary. This is not to say that I directly perceive this expression in the same way I experience joy first-hand. In the words of Gallagher and Zahavi, the expression is ‘saturated with the meaning of the mind; it reveals the mind to us’, and in this sense, we experience the emotion as directly as we can without first-person access (Gallagher & Zahavi, 2012: 207). So too, Jaspers’ empathic understanding allows for the ‘immediate grasp of expressive phenomena’ (Jaspers, 1968: 1317). In this sense, we can interpret Jaspers empathic understanding as quasi direct, thus retaining a distinction between I and Other. This would explain Jaspers ostensibly paradoxical description of empathic understanding as simultaneously direct and indirect.

The result of empathic understanding is more than a mere description of ‘what it is like’ to have a certain psychiatric condition; it is an attempt to gain an in-depth knowledge of the interpersonal, intentional, temporal, spatial and affective structure of the patient’s life-world. Jaspers describes empathic understanding as ‘spontaneous’ and ‘non-intellectual’(Jaspers, 1968: 1315). This has led some philosophers, such as Sass, to raise an inconsistency between the reflection Jaspers emphasises in empathic understanding and its ‘non-intellectual’ nature (Sass, 2013: 105). However, it seems that the spontaneous nature of empathic understanding is due to it being a habitualised action, that can only be employed pre-reflectively through careful training and repeated practice: ‘we need to train ourselves in it and master it’ (Jaspers, 1968: 1316).

Once empathic understanding has been mastered, Jaspers posits that the clinician obtains an inner representation of the patient’s experiences: ‘it is possible to partake in the inner life of another person through a tentative exchange of roles; a certain dramatic play…which nevertheless is no play but real’ (Jaspers, 1997: 21). In so doing, the patient and psychiatrist co-inhabit the same life-world (Miterleben). For Jaspers, this understanding is a form of perception as the psychiatrist does not merely think along with patient, but *sees* along with them: ‘The process is not only one of simple observation, like reading off a measurement, but the exercise of a self involving vision in which the psyche itself is glimpsed’ (ibid).

It is important to note that for Jaspers, ‘empathic understanding’ has its limitations. In *General Psychopathology*, Jaspers contrasts that which is ‘meaningful and allows empathy’ with the ‘un-understandable’ (Jaspers, 1997: 577). Falling into the latter category for Jaspers are the primary delusions of schizophrenia,

What is it about the nature of these primary delusions that defy the empathic approaches to understanding? For Jaspers, there is something about the uncanny nature of such experiences that prevents the psychiatrist from being able to relive it. Jaspers questions ‘how far can our understanding go in such cases, when we cannot base it on any conscious experiences of a similar kind’? (Jaspers, 1968: 1318). In addition, such experiences do not lend themselves to expression through interview or self-description; due to the ineffable nature of condition, psychosis often defies language (Jaspers, 1968: 1319)^10^. However, Jaspers does not suggest that the subjective symptoms of the person with primary delusions cannot be understood at all. For the ’ununderstandable’, Jaspers suggests we turn to ‘explanation’ or Erklaren as he believes that primary delusions can only be understood through naturalistic causal explanation rather than understanding them as meaningfully motivated by various circumstances.

The limitations of empathic understanding reveal further aspects of its phenomenological character. If empathy breaks down in a case of radically altered lived experience, empathic understanding necessitates a shared experience of the world between clinician and patient. This is reminiscent of Merleau-Ponty’s account of intersubjectivity, whereby he concludes that we have an intersubjective relationship with the Other as we recognise that we engage in a shared world. Merleau-Ponty provides an example where he observes a man who is impacted by the world in the same way as himself: the sun burns them, makes their eyes squint, makes them sweat, makes them raise their hand over their forehead in a protective gesture or reach for a hat (Merleau-Ponty, 1973: 136). Merleau-Ponty identifies in the Other’s gestures that they experience the same ‘bite of the world’ as himself (Merleau-Ponty, 1973: 137). In contrast, for Jaspers, those that experience a radically different ‘bite of the world’ cannot be understood through empathic methods^11^.

Jaspers observes three ways in which one can exercise empathic understanding. (1) immersion in the ‘gestures, behaviour, expressive movements’ of the patient; (2) exploration of the patient’s experience through structured interview; and (3) written self-descriptions (Jaspers, 1968: 1317). While Jaspers acknowledges that self-description is the hardest of the three to perform, it is the most valuable phenomenological method when successful. This is because self-description provides an unclouded, reliable account of a lived experience and does not risk falling prey to the prejudice that may imbue a psychiatrist’s questioning.

The expanding wealth of research and application of phenomenological psychopathology in recent years is a testament to Jaspers’ work, and to the value of a patient-centred approach to psychopathology. Jaspers has not only redefined the boundaries of what we call psychopathological knowledge, but has also brought to light the therapeutic and scientific benefits of an approach to psychiatric healthcare that focuses on the lived experience of the patient. Empathic understanding is a core part of Jaspers’ methodology, as it is the sole means by which the clinician accesses the subjective phenomena. Nevertheless, in what follows, we take issue with the concept of empathic understanding on epistemic and ethical grounds.

## Challenging empathic understanding

### Radical and second-order Empathy

Recall, Jaspers argues that those with radically different experiences are immune to empathic understanding. The only condition Jaspers identifies as ‘un-understandable’ are primary delusions in schizophrenia, which he describes as ‘a transformation in our very awareness of reality’ (Jaspers, 1997: 95). Indeed, there is a history of delusional experiences, and schizophrenia more broadly, being singled out as being profoundly different lived experienced. Contemporary phenomenological accounts of schizophrenia agree that those with the condition experience a dramatically altered lived experienced (Parnas et al., 2005; Henriksen, 2013; Sass, 2017; Krueger, 2020; Ritunanno, 2022). It has been observed that this makes schizophrenia particularly difficult to understand for the person who has not experienced it:

It is often difficult for a person with such a sense of his integral selfhood and personal identity, of the permanency of things, of the reliability of natural processes, of the substantiality of natural processes, of the substantiality of others, to transpose himself into the world of an individual whose experiences may be utterly lacking in any unquestionable self-validating certainties. (Laing, 2010: 39).

While, schizophrenia can be grasped through alternative methods, for Jaspers, empathic understanding is not up to the task of interpreting such a radically different lived experience.

The inclusion of the ‘un-understandable’ has provoked contemporary phenomenological psychopathologists to revise Jaspers’ account of empathic understanding. Ratcliffe (2012, 2013), Sass (2013) and Stanghellini (2013, 2018) argue that schizophrenia can be understood through an alternative form of empathy- a ‘radical empathy’ (Ratcliffe, 2013) or a ‘second-order empathy’. (Stanghellini, 2013). Both revisions of empathic understanding introduce an additional step to the procedure that allows one to effectively bridge the ‘unbridgeable’ (Sass, 2013: 106).

Sass, Stanghellini and Ratcliffe all advocate for a kind of empathic perception that is between direct and indirect perception of the Other, a ‘quasi-perception’ as Ratcliffe puts it. This quasi-perception seems to fit the Husserlian account of empathy with the Other, whereby one grasps the Other’s expression and experiences it quasi-directly, whilst still not as directly as first-person perception. In so doing, Ratcliffe claims we can form an ‘us’ experience, much like Jaspers’ notion of the Miterleben (shared experience) (Ratcliffe, 2012: 488). On these grounds, one can empathically understand even the most radically different lived experience through’ radical’ or’ second-order’ empathy. As noted in the previous section, we believe that Jaspers already had a quasi direct form of empathetic understanding in mind.

In addition, Ratcliffe and Stanghellini advocate for the use of the phenomenological reduction prior to empathy. In order to understand the ‘un-understandable’ through radical empathy, Ratcliffe suggests a suspension of the natural attitude: ‘If we know or suspect that the other person has never seen buses before and has no grasp of the norms associated with them, we might, when engaging with her experience, bracket our more usual assumption that these are features of a shared world’ (Ratcliffe, 2012: 478). So too, Stanghellini proposes: ‘Achieving second-order empathy thus requires bracketing my own pre-reflexive, natural attitude (in which my first-order empathic capacities are rooted), and approaching the other’s world as I would do while exploring an unknown country’ (Stanghellini, 2013: 169). This too is already a feature of Jaspers’ empathic understanding.

The key distinction between Jaspers’ empathic understanding and second-order empathy’ or ‘radical empathy, is that the latter call for an acknowledgement of the difference between I and the Other. Stanghellini states, ‘I must acknowledge the radical difference that separates me from the way of being in the world that characterises the other’ (Stanghellini, 2013: 169). Ratcliffe refers to this as adopting a ‘distinctive kind of other-directed attitude’ (Ratcliffe, 2012: 486). Adopting the ‘Other-directed attitude’ is an intellectual, theoretical endeavour which allows for an ‘openness’ to the Other (Ratcliffe, 2012: 486). Only upon recognising the profound difference of the Other’s life world can we have a ‘kind of dynamic, quasi-perceptual exploration of another person’s experience’ or a ‘phenomenological appreciation of their experience as it is for them’ (ibid).

While ‘radical’ or ‘second-order’ empathy is a promising development of Jaspers’ account, we argue that there are fundamental problems with empathic understanding that the evolved concepts fail to overcome. While we can indeed form an intersubjective connection with the Other who has a radically different life experience through a kind of ‘openness’ (in line with the accounts of intersubjectivity by Husserl, Heidegger, Merleu-Ponty to name a few), this does not necessarily entail ‘understanding’ the Other’s experience. In the words of Jaspers, ‘we should in no circumstances be content with a general impression extracted from the total picture’(Jaspers, 1968: 1323). More significantly, there are high risks of being overly presumptuous of one’s ability to *understand* the life-world of the Other through an empathic approach.

### Transformative experience

At this juncture, it is important to observe that in the psychiatric encounter, both clinician and patient bring essential knowledge to the table in order to understand and treat the condition in question. The clinician contributes expertise in psychiatric methodology, causal processes, treatment methods and so on. The clinical knowledge possessed by the healthcare professional should not be discounted or trivialised. However, this clinical knowledge should not be conflated with experiential knowledge. As previously stated, our interest here lies in clinicians who do not have the requisite lived experience of the condition in question and attempt to obtain it through empathic understanding.

Although Jaspers cautions against losing ‘the sense of the inexhaustibility and the enigma of each [person with a psychiatric condition], which we ought to keep in the face of the seemingly most trivial cases^12^’, empathic understanding ostensibly does just that (Jaspers, 1910: 85). We argue that to overstate the level of insight the clinician can gain from any form of empathic understanding can lead to a misunderstanding and a misappropriation of a psychiatric condition. Stanghellini claims ‘by unfolding the structures of a [a patient’s pathography], we can understand an author better than the author himself’ (Stanghellini, 2018: 962). However, assuming that the clinician can ‘know’ the lived experience of the Other risks an inaccurate representation of the subjective phenomena and risks undermining the patient’s epistemic privilege.

Psychiatric ‘illness’ is an epistemically ‘transformative experience’ (Paul, 2014). Due to a monumental shift in the person’s embodied experience, she is thrust into an unfamiliar life-world with new and often inexpressible meaning-structures. Such contexts ‘gives us experiences that we would not otherwise have had and that we cannot know what it is like to have until we undergo them—knowledge that cannot otherwise be acquired’ (Carel et al., 2016: 1152). In other words, certain experiences, such as childbirth or an ecstatic religious experience with a God, can only truly be understood by those who have had both ‘the requisite bodily experience’ (as in the case of childbirth) and, or the requisite interpretation of the world (for instance, an ecstatic religious experience requires an understanding of the world as one with a God) (Kidd & Carel, 2017: 185). Take depression; according to Styron, the incomprehension of depression by others is driven not by a lack of sympathy, ‘but the basic inability of healthy people to imagine a form of torment so alien to everyday experience’ (Styron, 2010: 14–15). This epistemically transformative experience, we suggest, makes empathic understanding impossible to perform for those without the requisite lived experience. Thus, in attempting to assume the first-person perspective of the patient, the clinician is likely to misrepresent the condition. Consequently, empathic understanding is highly vulnerable to error and hinders knowledge acquisition.

### Epistemic injustice

Second, there is a danger that empathic understanding could facilitate a unique form of epistemic injustice. Epistemic injustice was first theorised by Fricker to ‘delineate a distinctive class of wrongs, namely those in which someone is disingenuously downgraded and/or disadvantaged in respect of their status as an epistemic subject’ (Fricker, 2017: 53). The epistemic nature of the injustice derives from a person being wronged in their capacity as a knower, as someone who can convey knowledge. The injustice of epistemic injustice lies in the marginalised person being ‘degraded qua knower, and… symbolically degraded qua human’ (Fricker, 2007: 44). This is because an essential aspect of their humanity, their epistemic agency, is challenged.

For Fricker, epistemic injustice is necessarily non-deliberative. By this, she means epistemic injustice is not a calculated false representation of another person’s epistemic agency; its operations are far more implicit. When epistemic injustice occurs, the hearer is swayed by a deep-set identity prejudice they are unaware they even hold. This ‘absence of deliberate, conscious manipulation’ makes epistemic injustice so difficult to spot and so important to name (Fricker, 2017: 54). Fricker goes on to distinguish two forms of epistemic injustice: hermeneutical injustice (where certain groups are excluded from contributing the hermeneutical framework, resulting in gaps in our knowledge) and testimonial injustice (where testimony from marginalised groups are dismissed by virtue of an identity prejudice).

In comparison to alternative methods in psychiatry, phenomenological psychopathology has rightly been heralded as a methodology that champions epistemic *justice* (Kidd, Spencer, Carel, 2022), (Ritunanno, 2022), (Spencer, 2021). Rather than a one-size-fits-all approach, phenomenological psychopathology strives to facilitate reflective awareness and communicability of the patient’s first-person account through doctor-patient dialogue. In so doing, phenomenological psychopathology advocates for the epistemic agency of the patient through1) testimonial *justice*, as the first-person reports of the patient are taken seriously 2) hermeneutical *justice*, as phenomenological psychopathology rejects the interpretive framework normally advanced by diagnostic manuals, and instead aims to create a language that originates from the patient’s experience. However, as phenomenological psychopathology is upheld as a benchmark for epistemically just approaches to psychiatry, it is all the more important that we closely examine the methodology, especially those that are over 100 years old. In this section, we argue that one antiquated aspect of the methodology, that of ‘empathic understanding’, inhibits the epistemic agency of the patient and therefore needs to be replaced if epistemic justice is to be achieved.

Examining empathy in the medical encounter more broadly, Stefanello argues that epistemic injustice occurs through clinical empathy due to a general diminishment of the patient’s credibility, resulting from the epistemic power imbalance between patient and clinician (Stefanello, 2022: 492). Indeed, such an epistemic power imbalance in psychiatry is well documented in the literature (Kidd, Spencer & Carel, 2022). However, while there may be an overall unequal distribution of credibility, in the context of phenomenological psychopathology it is not immediately apparent what specific epistemic harms arise from empathic understanding. It does not seem to be testimonial injustice, as the patient’s testimony is carefully listened to, taken seriously and valued by the hearer. It does not seem to be a hermeneutical injustice, as the patient’s perspective directly informs how we come to understand a given condition in phenomenological psychopathology. As the clinician is attempting to draw out the knowledge of the patient, the patient is recognised as a knower.

The two forms of epistemic injustice we identify as resulting from ‘empathic understanding’ are more complex than those discussed thus far. The first is a form of Emmalon Davis’ ‘epistemic appropriation’ that we call ‘epistemic co-opting’. Here, the injustice lies in the clinician believing they have equal subjective knowledge to those with the requisite lived experience. Davis introduces the concept of ‘epistemic appropriation’ to capture not a ‘conceptual deficit’ to epistemic resources but instead ‘a sort of conceptual theft’ (Davis, 2018: 719). The theft, for Davis, involves a dominantly situated individual or group robbing a marginalised group of their cultivated epistemic resources. These epistemic resources are thus appropriated for their own gain without recognising the original epistemic contributors.

However, the epistemic appropriation described by Davis does not precisely fit the harm that we recognise in empathic understanding. The clinician acknowledges the source of understanding as stemming from the patient, and thus there is no ‘epistemic detachment’ or ‘epistemic misdirection’ (Davis, 2018). For this reason, ‘appropriation’ is not the correct term to use here. Instead, we identify the harm at play here to be some kind of conceptual ‘*co-opting’* (rather than theft). Therefore, we argue that if the clinician claims to have the same knowledge of the lived experience as the patient, they co-opt the patient’s ‘epistemic privilege’.

The term ‘epistemic privilege’ was popularised by standpoint feminist theory and originally pertained to the invaluable insight a woman has into her experiences of womanhood (Hartsock, 1983). Given this insight, standpoint theorists argue that women should be responsible for defining their experiences in their own terms. In this sense, the term privilege is not a beneficial social position that the identified group (women) have unduly bestowed on them. Rather it is a unique and vital perspective on a lived situation. The concept of epistemic privilege need not be so narrow, however. In *Black Feminist Thought*, Patricia Hill Collins establishes Black women’s standpoint on their experiences of family, friendship, communities, religion, and work (Collins, 1990). Collins emphasises the value of such lived experience, not only as a means to expand Black feminist knowledge and to challenge power dynamics. Collins hints that there is something inherently valuable about that knowledge itself, regardless of the secondary benefits. It is an essential aspect of the Self, being an ‘agent of knowledge’ through specific lived experiences (Collins, 1990: 266). Indeed, self-definition is an essential aspect of epistemic privilege; in the words of Bowell ‘This assertion of identity—of who I am—adds to a body of knowledge about how my life is and how I experience the world’ (Bowell, 2011). Asserting one’s standpoint is to assert one’s authentic identity and to transcend the stereotypes and prejudices manifested by more dominant groups.

By overstating the clinician’s ability to access the patient’s lived experience (their standpoint) through the empathic approach, the clinician risks ‘co-opting’ the epistemic privilege of the marginalised subject. As such, we contribute the term epistemic co-opting to the literature on epistemic injustice^14^. In doing so, the clinician co-opts something that essentially does not belong to them. They co-opt the lived experience integral to the identity and the self-definition of the patient, and claim some knowledge of it. They attempt to participate in a personal, identity informing process of self-assertion, despite not being a member of the marginalised group and not having the requisite lived experiences. This elicits an epistemic harm, as it dilutes the patient’s claim over their lived experience and their status as a self-defining knower is impugned.

In addition, epistemic co-opting may include what Alessandra Tanesini refers to as ‘intellectual arrogance’. Unlike in cases of ‘haughtiness’, intellectual arrogance does not ‘necessarily involve a sense of superiority or disrespect for other epistemic agents’ (Tanesini, 2016: 82). Tanesini captures ‘intellectual arrogance’ through the example of the scientist: ‘A scientist may be arrogant in the way in which he conducts his inquiry…He may, for instance, be unwilling to contemplate that he has made a mistake when an experiment produces results that are not credible’ (ibid). Tanesini recognises that intellectual arrogance may be motivated by a sense of unaccountability, and/or ‘an overestimation of one’s abilities’ (ibid). It is the latter that seems to occur in a case of epistemic co-opting, as the clinician assumes the possession of a knowledge that they cannot obtain^15^. As such, the clinician mistakenly inflates their own epistemic capacities.

The second epistemic harm we identify is epistemic objectification. Fricker recognises that one can be undermined as an epistemic agent not only by being perceived as a bad informant but also as a mere ‘source of information’. Unlike an informant ‘(as when someone tells one something one wants to know)’, a source of information conveys knowledge that the hearer can ‘glean’ (Fricker, 2007: 132). To describe ‘gleaning’ information, Fricker uses the example of inferring that it is raining from a guest who ‘arrives bedraggled and shaking her umbrella’ (ibid.). Fricker states that when a speaker is reduced to a source of information rather than an epistemic agent, they are subjected to epistemic objectification. Fricker identifies that even a speaking subject can be epistemically objectified, as she provides the example of the objectification of Tom Robinson by the jurors of Maycomb County in To Kill a Mockingbird^16^, where the members of the jury ‘radically undermine [Tom Robinson’s] general status as an epistemic subject’ (Fricker, 2007: 135).

Such objectification has been found to occur in the domain of psychiatry, most notably by Michel Foucault. Foucault recognised the objectification of the patient as a fundamental strategy of the 1960’s asylum, whereby the psychiatrist becomes the observer, and the patient is reduced to the observed. As ‘the observed’, Foucault argues that the voice of the psychiatric patient becomes inconsequential. Initially, this point seems not to pertain where the patient is directly addressed and their narrative plays a pivotal role in their psychiatric classification and treatment. However, for Foucault, such talking therapies are still a form of observation; in fact, he claims that psychoanalysis ‘doubled the absolute observation of the watcher with the endless monologue of the person watched’ (Foucault, 2001: 238). He suggests that the patient’s narrative is not truly speech expression, or at least not speech expression with any power. Instead, the ‘endless monologue’ elicits further behaviour from the patient to submit to psychiatric scrutiny, ‘thus preserving the old asylum structure of non-reciprocal observation but balancing it, in a non-symmetrical reciprocity, by the new structure of language without response’ (ibid.).

It would be a false analogy to liken the asylum of the 1960s or psychoanalysis to phenomenological psychopathology. However, Foucault’s thoughts on observation are not altogether anachronous, as empathic understanding risks treating the patient as merely a ‘source of information’. In the words of Jaspers:

Just as sense-perceptions are evoked by the demonstration of an object, so this meaningful empathic actualisation will be evoked in us by the above-mentioned hints and indications, by our immediate grasp of expressive phenomena and our self-immersion in other people’s self-description (Jaspers, 1968: 1317).

Although the patient speaks, their testimony risks being treated more like observable phenomena from which information can be gleaned. Stefanello raises a similar concern as she states: ‘the interpretative work of empathy seems to become an issue that is beyond the interest of the patient and becomes a concern exclusively for the doctor’ (Stefanello, 2022: 491). So too, in phenomenological psychopathology the patient appears somewhat passive as the clinician observes and interprets the subjective phenomena. Drawing on Fricker, it could be argued that the patient loses their epistemic subjectivity (Fricker, 2007: 135).

Objectification is a common complaint by people with who have experienced psychiatric healthcare:

If you enter the psychiatric business as a patient, then you have a high chance of being reduced to a disturbing object or to the disorder itself. Only that which is significant to the diagnostic examination is seen and heard. We are examined but not really seen; we are listened to but not really heard (Fusar-Poli et al, 2022).

While Jaspers describes the methodology the clinician must employ in great depth, the role of the patient is to simply recount their phenomenal experience as best they can. Indeed, Jaspers casts doubt on the patient’s abilities to observe and interpret their own subjective phenomena: ‘patients can rarely be induced to carry out self-observation, and then only in very favourable conditions, in regard to simple disturbances’ (Jaspers, 1968: 1317).

Modern concepts of ‘radical’ and ‘second-order’ empathy may curtail epistemic objectification, as the process of ‘openness’ towards the Other must necessarily recognise the Other as a Subject. However, in practice, the patient is still vulnerable to being relegated to a passive bystander as the clinician extracts the meaning structures of the patient’s experience from ‘the vantage point from which he sees the patient’s situation’ (Stanghellini, 2018: 962). The process often remains highly observational, especially as perception (whether quasi-perception or direct perception) is essential to empathic understanding. The Other is often portrayed as a being having their subjective phenomena observed and interpreted, rather than a talking, epistemic agent, communicating their subjective phenomena directly.

Consider Ratcliffe’s key example of the radical empathy experienced when reading a bedtime story to a young child: an ‘affective and gestural dialogue’ is formed as the child ‘giggles and points’, so that eventually ‘you share in his joy, his enthusiasm’ (Ratcliffe, 2012: 488). Interestingly, in this example, the Other’s subjective phenomena is observed through gesture, as opposed to verbal communication^17^. Thus, there is a danger of minimising the knowledge gained through the patient’s communications. Rather than observing, the clinician should reflectively listen to the patient’s first-person account of their phenomenological experience (we consider this in greater detail in the final section). The clinician can better grasp the subjective phenomena of a psychiatric experience if the patient is not relegated to an epistemic object.

Following this exploration of empathic understanding and its contemporary forms, we argue that ‘empathic understanding’ risks overstating one’s ability to directly access the patient’s experience. There is a danger of (1) error due to transformative experience (leading to misdiagnosis, mistreatment and an overall misunderstanding of the condition at hand) and (2) epistemic injustice, through co-opting the patient’s experience and intellectual arrogance (epistemic co-opting) and epistemic objectification. On these grounds, we argue that empathic understanding is an unhelpful concept in phenomenological psychopathology. Attempting to have a surrogate lived experience of a psychiatric condition only hinders understanding and risks undermining the epistemic agency of the patient. Rather than attempting to overcome the limits of empathic understanding, we are inclined to agree with Jaspers that it is impossible to reproduce a ‘radically different lived experience’ in the clinician and extend this idea beyond schizophrenia to encompass other diagnoses. This is not to say that it is impossible for the clinician to understand a given condition; rather, an alternative methodology is necessary. Ratcliffe, Sass and Stanghellini emphasise the importance of recognising the difference of the Other’s lived world experience. After acknowledging this, instead of attempting to reshape empathic understanding so the clinician can still have a quasi-direct perception of the patient’s experiences, we ought to respect this difference and face the fact that it means we cannot perceive their experience.

## Virtuous listening

In place of the concept ‘empathic understanding’, we propose a development of Fricker’s ‘virtuous listening’. Fricker defines virtuous listening as ‘a more pro-active and socially aware kind of listening’ that would help ‘generate a more inclusive hermeneutical microclimate’ (Fricker, 2007: 171). Much like Fricker, Stanghellini identifies the significance of ‘listening’ to mediate a therapeutically promising exchange:

it is important to note that this process of unfolding is profoundly rooted in hearing—or even better: listening and dialoguing… Hearing contributes to an ethics based on reciprocity and belonging, as well as to establishing a kind of knowledge focused on subjective experiences and personal narratives (Stanghellini, 2018: 960).

What is importantly distinct about virtuous listening however, compared to the methods proposed by Stanghellini and Ratcliffe, is that it does not presuppose that the clinician can access the patient’s lived experience in any way. Rather, only the patient has access to this experiential knowledge. The clinician and patient then combine their clinical and experiential expertise in order to collectively draw out the relevant meaning structures. This re-establishes the patient’s role as an epistemic agent, as their unique epistemic privilege is acknowledged, and they adopt an equal interpretive role. Such virtuous listening allows for a more accurate and epistemically just examination of the interpersonal, intentional, temporal, spatial and affective structure of the patient’s life-world.

We propose that virtuous listening has a phenomenological dimension. Rather than relying on models of intersubjectivity that focus on the I connecting to the Other through the observation of gestural behaviour, a more appropriate approach in this context would be Merleau-Ponty’s account of speech expression. In the words of Merleau-Ponty, ‘what justifies the special place that is ordinarily accorded language- is that, of all expressive operations, speech alone is capable of sedimenting and of constituting an intersubjective acquisition’ (Merleau-Ponty, 2012: 195–196). Upon listening to (or reading) the speech expression^20^ of the Other, Merleau-Ponty describes a ‘taking up’ of the Other’s speech expression within one’s bodily infrastructure: ‘there is a taking up of the other person’s thought, a reflection in others, a power of thinking according to others, which enriches our own thoughts’ (Merleau-Ponty, 2012: 184). By engaging with the speech expression of the Other, one forms with them a kind of intersubjective ‘social whole’ (Merleau-Ponty, 1973: 145)^21^.

This is particularly the case when the Other is performing what Merleau-Ponty calls ‘speaking speech’. Speaking speech, also known as ‘authentic’ speech’, is a spontaneous and creative speech-act of first-hand meaning-making. In a footnote, Merleau-Ponty exemplifies speaking speech through ‘the lover who discovers his emotion’, ‘the “first man who spoke”’ and ‘the writer and the philosopher who awaken a primordial experience beneath traditions’ (Merleau-Ponty, 2012: 184, n.7). These disparate forms of speaking tie together to capture the most original of speech acts, whereby the speaker says something altogether *new*. Speaking speech is an expressive act that contributes towards the meaning structures of the world. It calls forth new ways of understanding the world, both for the speaker and the hearer. We suggest that a natural addition to this list would be the person attempting to express a profoundly unusual experience, such as that of psychiatric ‘illness’.

Merleau-Ponty illustrates the impact of speaking speech from one person to another through the example of reading a ground-breaking piece of work:

I start to read the book idly, giving it hardly any thought; And suddenly, a few words move me, the fire catches, my thoughts are ablaze, there is nothing in the book which I can overlook, and the fire feeds off everything I’ve ever read. I am receiving and giving in the same gesture. (Merleau-Ponty, 1973: 11).

Here Merleau-Ponty makes it explicit that speaking speech has the potential to build upon the recipient’s thoughts and evolve them. Speaking speech injects these commonplace words with new life, creating original meaning structures in the recipient’s horizon. By reflectively listening to the patient, we argue that the clinician can better grasp the lived experience of the patient. This phenomenological account of virtuous listening can allow for the essential intersubjective connection with the patient in question, without suggesting that the clinician can transfer themselves into the patient’s life-world. Virtuous listening thus recognises the transformative experience of psychiatric ‘illness’, and avoids the error of assuming a knowledge that one cannot attain. The patient is necessarily a speaking subject, expressing authentic and creative ‘speaking speech’, thus avoiding epistemic objectification and epistemic co-opting.

In line with virtuous listening, we encourage a move towards co-production in phenomenological psychopathology. ‘Co-production’ in mental health research acknowledges the valuable knowledge and expertise of people with lived experience of psychiatric ‘illness’ or neurodiversity. It champions the production of joint research between experts by experience and academics/clinicians, who would contribute their insights equally. For example, recent co-production work in phenomenological psychopathology has been used to shed light on the core phenomena experienced in psychosis (Fusar-Poli, 2022). Co-production ensures virtuous listening from the offset. It establishes an equal epistemic agency between the academic, clinician and patient. Co-production thus avoids epistemic error, epistemic objectification and epistemic co-opting.

To be clear, we do not suggest that virtuous listening is not already practised in phenomenological psychopathology and psychiatry more widely- it certainly is. Many clinicians reflect on their position as a knower in comparison to their patients and listen carefully to the lived experience they contribute. Our concern is that there is a problem with an aspect of the *methodology* championed by phenomenological psychopathology- that of empathic understanding. We suggest that this aspect of the methodology is outdated and no amount of revision can save it from its ethical flaws. For this reason, we propose that empathic understanding ought to be swapped out for virtuous listening in the methodology of phenomenological psychopathology in order to avoid the epistemic and ethical problems it raises. Correcting this methodology aligns with the epistemically just ethos of phenomenological psychopathology.

## Conclusion

In the search for alternative approaches to psychiatry, there has been a reignited interest in recent years for phenomenological psychopathology (Stanghellini et al., 2018) (Stanghellini & Fuchs, 2013). Through this revival, it is essential that we take forward the most useful methods from the tradition and leave behind those that are prone to error and are ethically problematic. Promising as it may initially seem, we suggest that empathic understanding is an aspect of the methodology that ought to be jettisoned in favour of approaches that champion the epistemic agency of the patient. Through this paper we considered modern attempts to salvage the concept of empathic understanding, in particular radical empathy (Ratcliffe, 2012) and second-order empathy (Stanghellini, 2013). The main contribution of these forms of empathy in phenomenological psychopathology is the recognition of the difference of the Other. Yet, we demonstrate that, much like its predecessor, these modern adaptations of empathic understanding risk 3 key epistemic harms: (1) misunderstanding the lived experience of the patient, (2) co-opting the lived experience of the patient and (3) objectifying the patient themselves. By infusing Fricker’s account of virtuous listening with Merleau-Ponty’s phenomenology of speech expression, we devise a replacement for empathic understanding that reinstates the epistemic agency of the psychiatric patient.

## Data Availability

N/A

## Footnotes

1 Translation of quote found in Spiegelberg, 1972.

2 Jaspers second publication, a seminal piece on paranoic jealousy, was where he first explored this concept of empathic understanding (1910). This then went on to form a central theme in “The Phenomenological Approach in Psychopathology” (1968).

3 We acknowledge that the term ‘illness’, ‘disorder’ and ‘condition’ can be problematic, as it suggests that all forms of psychiatric ‘difference’ are necessarily pathological. We recognise the limits of this terminology.

4 This idea of healthcare as essentially hermeneutical persists in modern phenomenological psychopathology, taken up by Gadamer (1996), Svenaeus (2018) Stanghellini (2013).

5 It is important to note that General Psychopathology has been revised at several points during Jaspers’ life, from 1913 to when the 8th edition was published in 1965, and prior to Jaspers’ death in 1968. With these revisions, Jaspers’ ideas on genetic understanding likely evolved. His ideas on genetic understanding also evolved between the paper published in 1912 and the publication of General Psychopathology. As such, there is likely some inconsistency and evolution in Jaspers’ thought on genetic understanding. For the purpose of this paper, we present the account of genetic understanding that has the most uptake in the literature.

6 It is worth observing that Husserlian phenomenology refers to ‘genetic phenomenology’ (from 1917 onwards, so after Jaspers, 1968 essay, and the first edition of General Psychopathology) to understand the origin and history (‘sedimentation’) of the intentional structures of experience themselves (Donohoe, 2016:11).

7 For more on the relationship between genetic understanding and phenomenology see (Steinbock, 2018).

8 The first public reference to the phenomenological reduction can be found in Husserl’s lectures on ‘The Idea of Phenomenology’ (1907). Husserl eventually evolves this concept of the phenomenological reduction into the ‘epoche’, after the publication of Jaspers’ ‘General Psychopathology’ (1913).

9 Much like Jaspers, Husserl’s account of empathy also fluctuated between more direct and more indirect accounts of access to the expressive phenomena of the Other. See Zahavi (2014).

10 See also Sass (2017).

11 It is surprising that Jaspers’ account of the ‘un-understandable’ is limited to those who experience delusions, when many other psychiatric illnesses are described as radical transformations of one’s experience of the world. This radical alteration has been observed in depression (Ratcliffe, 2015) anxiety (Aho, 2020) and agoraphobia (Trigg, 2018).

12 As translated in Spiegelberg, 1972: 181.

14 Epistemic co-opting could occur outside of ‘empathic understanding’ and psychiatry in general. An example could be found in the speech given by the Fifa president, Gianni Infantino, in November 2022. In this speech, Infantino claimed that he felt ‘gay’, ‘disabled’ ‘like a woman’ and other marginalised groups (The Independent, 2022). Infantino’s point seemed to be that because he has experienced some forms of discrimination (he claimed to have been bullied as a child for his red hair and freckles) that he could understand the unique injustices felt by other marginalised groups. Therefore, this case of epistemic co-opting is not unique to phenomenological psychopathology. Future work is needed to identify epistemic co-opting outside of psychiatric healthcare.

15 The authors are grateful for Dr. Ian James Kidd’s fruitful insights on hubris and arrogance as an epistemic vice.

16 Berenstain criticises Fricker’s choice of fictionalised accounts to depict epistemic injustice against marginalised groups over the numerous real world examples she could draw upon. See (Berenstain, 2020: 749 n30).

17 To clarify, we do not deny the importance of understanding through non-verbal expression. This can be an essential means of understanding in instances where patients are non-verbal (See Spencer, 2022). What we hope to emphasise here is that when a patient is communicating verbally, it is important to take seriously their verbal communications.

20 Merleau-Ponty uses the term ‘authentic’ speech’ to describe a spontaneous and creative speech-act of first-hand meaning-making (Merleau-Ponty, 2012: 184). The creative speech acts that come from a patient trying to put into words their ineffable illness experience would qualify as an ‘authentic’ speech expression.

21 In the case of psychiatric illness, we do not deny that such ‘taking up’ is no easy task; psychiatric illness is characteristically ineffable and as such, patient’s frequently struggle to put these experiences into words (see Spencer, 2022). However, this does not mean uptake is impossible.
